# Parkinson's Disease Caregiver Strain in Singapore

**DOI:** 10.3389/fneur.2020.00455

**Published:** 2020-06-23

**Authors:** Siok-Bee Tan, Allison F. Williams, Eng-King Tan, Richard B. Clark, Meg E. Morris

**Affiliations:** ^1^Nursing Division, Singapore General Hospital, Singapore, Singapore; ^2^School Health Sciences, The University of Melbourne, Melbourne, VIC, Australia; ^3^School of Nursing and Midwifery, Faculty of Medicine, Nursing and Health Sciences, Monash University, Melbourne, VIC, Australia; ^4^Department of Neurology, National Neuroscience Institute (Singapore General Hospital Campus), Singapore, Singapore; ^5^Victorian Rehabilitation Centre, Healthscope ARCH and La Trobe Centre for Sport and Exercise Medicine Research, La Trobe University, Melbourne, VIC, Australia

**Keywords:** caregiver, carer, Parkinson's disease, well-being, quality of life, rehabilitation

## Abstract

**Background:** Caregiver strain is recognized globally with Parkinson's disease (PD). Comparatively little is understood about caregiver burden and strain in Asia.

**Objective:** To investigate caregiver strain for families living with PD in Singapore, in light of international data.

**Methods:** Ninety-four caregivers were recruited via people living with idiopathic PD in Singapore. Caregiver strain was assessed using the Zarit Burden Interview (ZBI); health status was assessing using the Cumulative Illness Rating Scale for Geriatrics (CIRS-G). PD disability measures were the Unified Parkinson's Disease Rating Scale (UPDRS) and modified Hoehn and Yahr (1967) Scale.

**Results:** Primary caregivers of people living with PD in Singapore were mostly cohabiting spouses, partners or offspring. Around half employed foreign domestic helpers. Mean caregiving duration was 5.9 years with an average of eight hours per day spent in caregiving roles. Most care providers were comparatively healthy. Caregivers reported significant levels of strain which increased with greater level of disability (*r* = 0.36, *n* = 94, *p* < 0.001). Associations were significant between caregiver strain and scores on the UPDRS mentation, behavior, and mood subscales [*r* = 0.46, *n* = 94, *p* < 0.001, 95% CI (0.28, 0.60)]. High scores on the UPDRS activities of daily living subscale were associated with caregiver strain [*r* = 0.50, *n* = 94, *p* < 0.001, CI (0.33, 0.64)].

**Conclusion:** Most caregivers in this Singapore sample reported high levels of strain, despite comparatively good physical function. Caregiver strain in PD spans geopolitical and cultural boundaries and correlates with disease severity. These results support the need for better early recognition, education, and support for caregivers of people living with PD.

## Introduction

Parkinson's disease (PD) is a debilitating and progressive condition that impacts the lives of individuals and their families (1). Although caregiver burden associated with PD is well-documented for Europe (2–7) and North America (8–11), there is a lack of published data for the south-east Asia region. Most elderly people with PD in countries such as Singapore live at home (12, 13). It is important to understand how PD affects their caregivers because the prevalence of PD in South-East Asia ranges from 79 to 193 per 100,000 population (14). More than six million people will be living with PD in Asia by 2030 due to rapid population aging and lifestyle factors (15). People with PD experience movement disorders, falls, and non-motor symptoms that can reduce mobility and quality of life (1, 16). As the disease progresses, it can place a heavy burden on primary caregivers (17, 18). The individual and societal costs of PD are substantial (8, 19–23), yet are not fully understood for Asia.

With PD progression, caregiving can sometimes be perceived as the main role of some family members (24, 25). Studies in Australia (19, 26), Europe (6), and the USA (8) have reported considerable caregiver burden associated with PD. Caregiver burden refers to the negative physical, mental, and socioeconomic sequelae associated with caring for a person living with a disability (27). Martinez-et al. (28) and Kelly et al. (29) reported associations between PD caregiver burden and caregiver health-related quality of life (HRQoL). Caregiver HRQoL as measured on the EuroQoL has also been shown to correlate with burden, as measured on the Zarit Carer Burden Inventory (ZBI) (*r* = −0.33 to −0.49, *p* < 0.01) (28). Studies in western societies have also reported that psychological aspects of caregiver strain are associated with the level of PD disability (3). For example, disability in PD measured by the Barthel Index was associated with increased caregiver burden (*r* = 0.46–0.53, *p* < 0.01) (17). Many of the studies in the literature have focused on US and European populations with comparatively low ethnic and cultural diversity (30). The results for PD caregivers and care recipients might not be generalizable to south-east Asia, where there are geographical, cultural, and geopolitical differences. For example, Tan et al. (31) found that overall PD incidence rates were comparable between Singapore and the West, yet there were significance differences in the inter-ethnic incidence rates of PD between Chinese, Malays, and Indians.

The current study aimed to increase understanding of the dimensions of caregiver strain and burden in Singapore and to consider the findings in the context of global reports. We also aimed to determine the factors associated with strain in caregivers of this sample of people living with this progressive neurological condition.

## Method

We conducted a cross-sectional survey with a convenience sample of 94 caregivers of individuals with PD. Patient data was also collected through associated caregivers. Recruitment was via a neurology specialist outpatient clinic at an acute tertiary hospital and a PD society in Singapore. For a 2-tailed bivariate analysis test with power of 0.80 and statistical significance at 0.05, a sample size of 85 care providers was required to detect a moderately strong relationship between variables (32). Criteria for the selection of caregiver participants included: (i) above 21 years of age; (ii) primary caregiver of a patient (care recipient) diagnosed with idiopathic PD by a neurologist; (iii) providing at least 3 h of daily care for 6 months or more; (iv) able to understand spoken English. The research protocol was approved by institutional review boards of The University of Melbourne (Ethics ID: 0719562) and Singapore General Hospital (Ethics ID: 2008/122/A). All subjects gave written informed consent in accordance with the Declaration of Helsinki.

A structured questionnaire was administered to the caregiver participants. It contained items on (i) care recipient and caregiver sociodemographic characteristics; (ii) health information about the care recipients, measured using the modified Hoehn and Yahr Scale (HY) (33), and the Unified Parkinson's Disease Rating Scale (UPDRS) (34); (iii) information on the burden and health status of the caregivers quantified by the ZBI (35) as well as the Cumulative Illness Rating Scale for Geriatrics (CIRS-G) (36).

The ZBI has 22 items relating to the impact of care-recipient disabilities on caregiver physical and emotional health, and social, and financial distress. The ZBI score sums individual items (range 0–88), with a higher score indicating greater caregiver personal strain or role strain (35). The maximum possible scores are 24 (six items) for personal strain and 48 (12 items) for role strain. The ZBI has previously been shown to be a valid and reliable instrument for caregivers with dementia in Singapore (37). We also used the CIRS-G to estimate medical and psychiatric multi-morbidity burden in care providers (38). This scale rates the severity of problems as mild (1), moderate (2), severe (3), or extremely severe (4) (36).

When applicable, Spearman's rank correlation coefficients (*r*) were calculated to assess the direction and magnitude of the associations between variables. The strength of the relationships was interpreted following Cohen's guidelines (39); relationships were deemed as small where correlations ranged from *r* = 0.10–0.29; medium when *r* = 0.30–0.49 to large *r* = 0.50–1.00. Multiple regression analyses were used to explore the relationships between caregiver coping and well-being with sociodemographic, caregiving, and disease severity variables. Data were entered and analyzed using Predictive Analytics Software (PASW) Statistics 18. All reported *p*-values were 2-tailed with alpha set at 0.05.

We also conducted a comprehensive literature search of global PD caregiver burden on PubMed (1 April 2020) using the terms “Caregiver burden/strain AND Parkinson's disease” where studies included both caregivers and PD participants. Initially, the titles were screened by two people for keywords. The abstracts were then screened to ensure that studies were conducted on PD and caregiver strain or burden. Full-text articles 2018-2020 were reviewed to extract data on caregiver burden inventory utilized, caregiver demographics/duration, and descriptive statistics (e.g., mean, median) reported for the caregiver burden inventory utilized in the studies.

## Results

### Parkinson's Disease Care Recipient Profile

Of the 94 Singaporean care recipients, 60 (64%) were male. The majority of the care recipients with PD were 51 years or older (*n* = 88, 93.6%). Six were aged 31–50 years. The age at which the care recipients were diagnosed with PD ranged from 33 to 89 years (*M* = 61.8, *SD* = 11.8). The mean duration of PD was 6.9 years (*SD* 5.6). Their mean UPDRS mentation, behavior and mood score was 3.4 (*SD* 2.9). The mean UPDRS ADL score was 15.4 (*SD* = 9.5). People living with PD had a range of levels of disease severity when functioning at their best (Table 1). It is notable that despite being rated five on the modified Hoehn and Yahr scale, 14% people with PD in the sample were being cared for at home.

**Table 1:** Hoehn and Yahr (33) stage when functioning at best.

	**Number**	**Percent**
Stage I Unilateral disease	19	20.2
Stage II Bilateral disease, without impairment of balance	8	8.5
Stage III Mild-moderate bilateral disease with some postural instability; physical independence	42	44.7
Stage IV Severe disability; still able to walk or stand unassisted	12	12.8
Stage V Wheelchair bound or bedridden unless aided	13	13.8

### Caregiver Profile

Most caregivers were females (*n* = 74, 78.7%). They were mainly spouses, partners, or daughters who lived with the people who had PD. The mean number of caregiving years was 5.9 years (range 0.25–25, *SD* 5.2). The care providers dedicated a mean of 8 h per day (*SD* = 7.0) in their PD caregiving roles. As shown in Table 2, foreign domestic helpers were often engaged to assist with personal care, rehabilitation, social engagement, and mobility (30). Notwithstanding, 58% of carers preferred help from family and friends, who were mainly siblings and sons, and daughters of care recipients. Approximately a quarter of the caregivers attended caregiver education or support groups to obtain more information about caring and rehabilitation for individuals with PD. Only a small percentage of caregivers attended more than 1 h of caregiver education and support.

**Table 2:** Caregiver demographics and characteristics of caregiving.

**Caregiving characteristics**		
**Demographics/ characteristics**	***n***	**%**
Gender (%)		
Female	74	78.7
Relationship with care recipient		
Spouse/Partner	44	46.8
Sibling	4	4.3
Daughter	29	30.9
Son	9	9.6
Friend	3	3.2
Other	5	5.3
Living with care recipient		
Yes	79	84.0
No	15	16.0
Domestic helper		
Yes	43	45.7
No	51	54.3
Other helpers	
Yes	57	60.6
No	37	39.4
Who assists?^a^		
Spouse/partner	9	15.5
Sibling	22	37.9
Daughter	6	10.3
Son	12	20.7
Other	9	15.5
Resources other than family/friends for support		
Yes	19	20.2
No	75	79.8
Caregiver education and support group		
Yes	25	26.6
No	69	73.4
Hours of caregiver education		
0 h	79	84
>1 h	15	16
Hours of support group		
0 h	74	78.7
>1 h	20	21.3

a *n = 58 (a = caregivers who received assistance from others beside the domestic helpers)*.

### Caregiver Strain in Singapore

Caregivers in this Singapore sample reported a mean ZBI score of 23 out of 88. The personal strain and role strain domains are shown in Table 3. In order to interpret the relative intensity of burden experienced by caregivers of people living with PD, the following cut-off values were used: 0–20 little strain; 21–40 mild to moderate strain; 41–60 moderate to severe strain; 61–88 severe strain (35). Some caregivers (*n* = 45, 47.9%) reported little burden; 37 caregivers (39.4%) had mild to moderate burden and 11 caregivers (12.8%) reported moderate to severe levels of burden.

**Table 3:** Zarit burden interview mean scores.

	**Mean**	**SD**	**Minimum**	**Maximum**	**Median**
Personal strain	13.0	6.8	0.0	37.0	13.5
Role strain	5.3	4.4	0.0	18.0	4.0
Total score	23.0	13.2	1.0	71.0	21.5

### Caregiver Multi-Morbidities

Most care providers were older women who were comparatively healthy, although some reported a range of health problems and multi-morbidities of mild to moderate severity as measured by the CIRS-G. The mean score for CIRS-G was 2 (*SD* = 2) and the CIRS-G Severity Index mean was 0.12 (*SD* = 0.15). In this sample, there were more healthy caregivers than those with multi-morbidity.

### Relationships Between Care Recipient Disease Severity and Caregiver Strain

There was a statistically significant, moderately strong positive correlation between modified HY scores, and caregiver burden when the people with PD functioned at their best (*r*_s_ = 0.36, *n* = 94, *p* < 0.001). Statistically significant moderate positive correlations were also obtained between the HY scores and caregiver burden when the people with PD functioned at their worst (*r*_s_ = 0.38, *n* = 94, *p* < 0.001, 95% CI [0.19, 0.54]). This was particularly notable for the UPDRS Mentation, Behavior, Mood score (*r*_s_ = 0.46, *n* = 94, *p* < 0.001, 95% CI [0.28, 0.60]). The relationship between UPDRS Activities of Daily Living score and caregiver burden also showed a strong positive correlation (*r*_s_ = 0.50, *n* = 94, *p* < 0.001, CI [0.33, 0.64]). The personal strain and role strain for care providers increased with PD severity.

### Global Analysis of Caregiver Strain

Table 4 summarizes data from our evaluation of key recent international studies of PD caregiver strain. The results show world-wide data demonstrating that care-giver strain is challenging and common, with many shared features between south-east Asia and other regions of the globe, as seen in the world-wide literature (40–79).

**Table 4:** Summary data from key recent international PD studies of caregiver strain.

**Lead author, year**	**Country**	**Key findings**	**Caregiver**	**Caregiver scales and values**			
				**Questionnaire/index and domain**	**Mean (*SD*)**	**Median/** **IQR/** **Range**	**Correlations** ***r***
			***n***				
			**Sex**				
			**Age (yrs), Mean (*SD*)**				
Balash, 2019	Israel	Progression of PD can affect male and female CG differently; some females experienced more stress	*n* = 122	Multi-dimensional Caregiver Strain Total Physical strain	24.2 (14.9), *f* 15.4 (15.4), *m*	NR	NR
Bartolomei, 2018	Italy	Sleep quality in PD was associated with caregiver burden and quality of life	*n*= 57 21 *m*, 36 *f* 62.0 (12.0)	Caregiver Burden Inventory	9.0 (12.5)	NR	NR
Carrilho, 2018	Brazil	Optimization of availabletreatment, with better control of PD severity, can decreaseburden among caregivers	*n* = 21 80% *f* 53 (12.4)	Zarit Burden Interview UPDRS	28		0.48 (*p* = 0.026)
Crespo-Burillo, 2018	Spain	Deep brain stimulation was not associated with lower caregiver burden. Apathy in PD was associated with caregiver overload	*n* = 11 66.0 (9.9)	Zarit Caregiver Burden Inventory	48.6 ± 17.8	NR	ZBI and UPDRS-III 0.46
Dahodwala, 2018	USA	Caregiver strain was related to PD severity	*n* = NR	Multidimensional Caregiver Strain Index	19.9 (16.7), Men 16.4 (15.1), Women	NR	MCSI & co-morbidity 0.33
Drexel, 2019	Germany	Caregiver burden was noted for some dystonia patients. A small proportion of caregivers had burden	*n* = 93 34 *f* 61.6 (13.5)	Caregiver Burden Inventory and Hours of Caregiving	8.6 (9.6)	0-48	Caregiving hours per day (*r* = 0.409, *p* = 0.001)
Genç, 2019	Turkey	Almost half of caregivers showed burden. No significant differences between burden experienced in caregivers of early-stage and late-stage PD.	*n* = 74 (G1 40 G2 34) G1 *f* 25 (62.5%) G2 *f* 26 (76.5%) G1 6.65 (15.75) G2 49.41 (14.32)	Zarit Caregiver Burden Inventory	Little or no burden, G1 = 21 (52.5) G2 = 18 (52.9) Mild to moderate burden G1 = 15 (37.5) G2 =12 (35.3) Moderate to severe burden G1 =2 (5.0) G2 =3 (8.8) Severe burden G1 =2 (5.0) G2 = 1 (2.9)	NR	NR
Henry, 2020	USA	Difficulties with mobility, emotional well-being, and non-motor symptoms of PD were predictors of reduced caregiver QOL.	*n* = 181 77.9% *f* 64.04 (10.50)	PDQ-Carer	CG QOL items	NR	
					Self-care		0.76
					Anxiety/depression		0.78
					Activities		0.80
					Mobility		0.40
					ADL		0.39
					Emotions		0.38
					Cognition		0.36
					Bodily discomfort		0.25
					Non-motor		0.42
Karlstedt, 2019	Sweden	Cognitive decline and poor ADL in PD was associated with CG burden	*n* = 22 *f* 70.7 (9.3)	Caregiver burden scale	42.5 (15.8)	NR	NR
Klietz, 2020	Germany	Motor UPDRS scores and patient's attentional symptoms were associated with caregiver burden	*n* = 78 64.8 ± 11.0	German version Parkinson's disease caregiver burden questionnaire	36.5 (27.1)	Range 0–100	PDQ-8 (*p* 0.064) and UPDRS part III (*p* 0.086) showed a trend toward association with caregiver burden
Kumar, 2019	Pakistan	Overall 62.8% caregivers were stressed; increasing stress and depression was related to PD progression. Most (86.7%) of the stressed caregivers were female (*p* < 0.0001)	*n* = 156 49 (31.4%) *f* 47.75 (11.98)	Caregiver Burden Inventory	NR	NR	NR
Lee, 2019	Korea	CB associated with higher daily time in caregiving. Better understanding of PD in spouses correlated with less burden	*n* = 142 72 *m*, 70 *f* 59.4 (14.4)	Caregiver Burden Inventory	52.0 (19.9)	49.0 (range 25.0–111.0)	CBI and: daily care time: 0.41 (*p* = 0.000) Care duration: 0.19 (*p* = 0.024)
Macchi, 2020	USA	Patient quality of life, anxiety and depression, and caregiver spiritual well-being contribute to caregiver burden	175 131 *f* (73%) 66 (11)	Zarit Burden Interview PDQ-39 PD QOL-AD Caregiver Reported HADS Anxiety Depression FACIT-SP spiritual well-being of caregivers	17.4 (7.9) 58.1 ± 28.4 33.8 ± 6.0 7.4 ± 3.6 4.3 ± 3.1 33.5 ± 8.7	NR	0.161 (*p* = 0.0001) 0.088 (*p* = 0.0001) 0.062 (*p* = 0.0014) 0.024 (*p* = 0.038)
Mosley, 2018	Australia	Caregiver burden was unchanged after subthalamic deep brain stimulation	*n* = 64 48 *f*, 16 *m* 58.3 (8.4)	Zarit Burden Inventory	21.4 (13.7)	21	NR
Rajiah, 2017	Malaysia	QoL domains such as “stigma” and “emotional well-being” in PD were associated with caregiver burden	*n* = 132 43 *m*, 87 *f* 45.1 (10.2)	Zarit Burden Interview	55.0 (19.2), mobility 39.0 (18.6), ADL 61.0 (17.4), emotional well-being 66 (16.8), stigma	NR	0.41
Smith et al., 2019	Mexico and America	Showed associations between PD-related impairments, caregiver burden, and caregiver mental health. Caregiver burden mediated the relation between PD-related impairments and caregiver mental health	*n* = 253 68.6% *f* USA group 76.4% *f* Mexico group 68.73 (8:36)	Short version Zarit Burden Inventory	NR	NR	NR
Tan, 2019	Singapore	Caregiver burden was associated with more prolonged disease, higher levodopa doses and motor fluctuations	*n* = 104	Zarit Burden Inventory	24.6 (15.3)	NR	NR
Tan, 2020 (this paper)	Singapore	Caregiver burden associated with PD disability, UPDRS mentation, behavior and mood subscale, and high scores on UPDRS ADL subscale	*N* = 94 caregiver-PwPD dyads caregivers: 74 f	Zarit Burden Inventory	23.0 (13.2)	NR	ZBI and HY 0.34 (at best) 0.35 (at worst) ZBI and UPDRS 0.38 (mood) 0.50 (ADL)
Trapp et al. 2018	Mexico	Caregiver burden mediated the relationship between family cohesion and quality of life	*n* = 95 78 *f* 51.1 (13.9)	Zarit Burden Interview	Family cohesion 24.26 (14.62)	0–70	−0.38
Tessitore, 2018	Italy	Statistically significant predictors of CB were caregiver need to change work and judgement of QoL. CB lower when treated with levodopa/carbidopa intestinal gel than continuous subcutaneous apomorphine or usual care	*n* = 126 57.9 (12.9)	Zarit Burden Inventory	LCIG−29.6 (14.4)		Work change: 23.6, 95%CI 10.4–36.8; *P* = 0.001
Torny, 2018	France	The severity of non-motor signs, patients' and caregivers' mood, and motor disease severity are the main determinants of caregiver burden	38 84% *f* 67.8 (9)	Zarit Burden Inventory PDQ-8 PD NMSS (total) HADS Depression	14.4 (12.7) 8.5 (6) 38 (41.6) 7.2 (4) 3.4 (4.2)		0.27 (*p* = 0.007) 0.46 (*P* < 0.0001) 0.16 (*p* < 0.0125) 0.35 (*p* < 0.0001)
Vatter et al. 2018	United Kingdom	Factor analysis revealed five burden dimensions (factors): 1. social & psychological constraints, 2. personal strain, 3. personal life, 4. concerns about future and 5. guilt. Factors were associated with lower relationship satisfaction, mental health, resilience, stress, anxiety, depression, resentment, negative strain, and PD motor severity.	*n* = 127 85.3% *f* 69.44 (7.62)	Zarit Burden Interview	35.51 (15.4)	35.00 2-74	factors 1 and 2 (*r* = 0.67), factors 2 and 3 (*r* =.67), factors 1 and 3 (*r* =.64) factors 3 and 4 (*r* =.62)
Yang, 2019	China	Caregiver self-efficacy mediated caregiver burden, caregiver anxiety and depression. Caregiver burden was related to poor cognition and poor motor function in people with PD.	*n* = 112 66 (58.9%) *f* 52.33 (13.43)	Chinese version of Zarit Burden Interview	19.58 ± 13.09	18.0 (0,59)	Caregiver burden & PD cognition −0.412 PD motor function 0.334

*f, female; m, male; NR, not reported; CG, caregiver; PD, Parkinson's disease; PwPD, People living with PD; LCIG, levodopa/carbidopa intestinal gel; ADL, Activities of Daily Living; G, group*.

*Instruments: PDQ-39, Parkinson's Disease Questionnaire; MCSI, Multidimensional Caregiver Strain Index; CSI, Caregiver Strain Index; CBI, Caregiver Burden Inventory; ZBI, Zarit Burden Interview/Zarit Caregiver Burden Inventory; CSI, Caregiver Strain Index; PDCB, Parkinson's Disease Caregiver Burden Questionnaire; UPDRS, Unified Parkinson's Disease Rating Scale; HY; modified Hoehn & Yahr Scale; MMSE, Mini-Mental State Examination; (HR)QoL, (Health-related) Quality of Life*.

## Discussion

Regardless of geographical, cultural, and geopolitical differences, caregiver strain in PD is a major problem world-wide and correlates with disease severity (40–79). Our study showed that most south-east Asian PD caregivers are family members. Although many received support from domestic workers, they often experienced high levels of burden and strain. Both role strain and physical strain were reported, warranting consideration of systems to be put in place for early recognition, education, and support for caregiver strain.

Our results are consistent with European (8) and American literature [eg., (28, 40, 41)] showing more female than male PD care providers, given that Parkinsonism more often affects men. Previous studies have also shown close relationships between disability and quality of life in people with PD and the level of burden in caregivers (42). Caregiver strain is particularly associated with immobility in PD care recipients and the severity of non-motor symptoms associated with PD (7, 43). A Malaysian study by Razali et al. also found that patient age, stage and severity of illness were significantly associated with feelings of burden in caregivers (44). However, caregiver burden in this Malaysian study was not related to social status, kinship or duration of care (44).

Approximately one quarter of the care providers in our study attended caregiver education or support groups to obtain more information on how best to support people living with PD. This was rarely more than an hour in duration. The small amount of PD education concurs with the findings of Mehta et al. who reported that caregivers who lived in Singapore often did not receive very much formal training to care for people living with PD (45).

The caregivers in our sample reported more personal strain than role strain. Overall, they exercised a reasonable level of control over their caregiving roles even though it sometimes affected their personal health and social life. This was consistent with regional studies showing associations between caregiving and perceived burden (23, 43, 46). For example, a Singapore qualitative analysis (47) reported that many caregivers experienced lifestyle restrictions and felt physically and emotionally drained. Others (24, 48–50) reported that increased involvement in caring can sometimes be perceived to disrupt a caregiver's personal life and roles. Mehta noted that some (but not all) Singaporean caregivers interpreted their role to be obligatory “having no choice (but to care for the patient)” (45).

The results of our investigation also showed that caregiver burden had a moderately strong positive relationship with PD disease stage (Table 4). It also escalated with severe disability. This finding is consistent with global reports (Table 4). Of interest, a Malaysian study reported that respite in the form of caregiver support group or day care attendance for patients can reduce caregiver burden (44). Thommessen et al. (51) and Schrag et al. (43) found that only the mental health of care recipients was associated with caregiver burden. In contrast, we found that both poor mental health and activities of daily living were associated with caregiver strain.

There was no correlation between caregiver multimorbidity and perceived caregiver burden in our sample. This was in contrast to Martinez-Martin et al. (2) who found that Spanish caregivers tended to be less physically and mentally healthy compared to the general population. The care recipients in our study had a higher disease severity than counterparts in Spain (12). Differences in race, culture, and healthcare systems might also have contributed to the disparate results.

Although this is the largest PD caregiver study undertaken in the Asia-Pacific region to have identified critical elements in caring, it is not known whether the Singapore results generalize to other locations in Asia such as Indonesia, Malaysia, Vietnam, Thailand, Cambodia, or China. The sample mainly included men with PD and caregivers who were women. Further clarification of gender issues in caregiving is warranted. The sample size for each ethnic group also did not allow for critical analysis of differences in caregiving as a result from race, ethnicity, and culture that could have affected caregiving experiences. In addition, variations in treatment options for different countries could affect caregiver burden and strain. Longitudinal changes in caregiver burden and coping strategies were not examined. Further trials are needed to better understand how caregiver strain varies according to the stage of progression of Parkinsonism as well as the effects of different interventions to improve health, well-being and quality of life in care providers and care-recipients.

## Conclusion

As with international data, care providers of people living with PD in Singapore often had raised levels of personal strain and role strain that were associated with the level of PD disability. The caregivers in our sample provided care for an average of 8 h per day for 6 years or more. Although foreign employed workers often gave assistance, around 80% of care providers were family members. Many care providers were elderly spouses. In Singapore, as for throughout the world, there is a need for systems to reduce caregiver burden that are responsive to the progressive trajectory of this common and chronic neurological disease.

## Data Availability Statement

The datasets generated for this study are available on request to the corresponding author.

## Ethics Statement

The studies involving human participants were reviewed and approved by the research protocol was approved by institutional review boards of The University of Melbourne (Ethics ID: 0719562) and Singapore General Hospital (Ethics ID: 2008/122/A). The patients/participants provided their written informed consent to participate in this study.

## Author Contributions

S-BT, AW, E-KT, and MM: contributed to research project conception and design. S-BT: original concept and research project organization and execution, data collection, statistical analysis, design and execution, and manuscript writing. AW: statistical analysis design, review, and critique. E-KT: statistical analysis review and critique, and manuscript: review and critique. MM: research project conception and design, and statistical analysis review and critique, and manuscript: review and critique. All authors contributed to manuscript revision, read and approved the submitted version.

## Conflict of Interest

The authors declare that the research was conducted in the absence of any commercial or financial relationships that could be construed as a potential conflict of interest.
